# Development of a new virtual reality test of cognition: assessing the test-retest reliability, convergent and ecological validity of CONVIRT

**DOI:** 10.1186/s40359-020-00429-x

**Published:** 2020-06-12

**Authors:** Ben Horan, Rachael Heckenberg, Paul Maruff, Bradley Wright

**Affiliations:** 1 School of Engineering, Deakin University, Geelong, VIC 3216 Australia; 2 School of Psychology and Public Health, La Trobe University, Wodonga, Victoria 3690 Australia; 3 The Florey Institute, The University of Melbourne, Parkville, VIC 3052 Australia; 4 School of Psychology and Public Health, La Trobe University, Bundoora, Victoria 3086 Australia

**Keywords:** VR, Eye-tracking, Concussion, Mild traumatic brain injury, Assessment

## Abstract

**Background:**

Technological advances provide an opportunity to refine tools that assess central nervous system performance. This study aimed to assess the test-retest reliability and convergent and ecological validity of a newly developed, virtual-reality, concussion assessment tool, ‘CONVIRT’, which uses eye-tracking technology to assess visual processing speed, and manual reaction time (pushing a button on a riding crop) to assess attention and decision-making. CONVIRT was developed for horse jockeys, as of all sportspersons, they are most at risk of concussion.

**Methods:**

Participants (*N* = 165), were assessed with CONVIRT, which uses virtual reality to give the user the experience of riding a horse during a horserace. Participants were also assessed with standard Cogstate computer-based concussion measures in-between two completions of the CONVIRT battery. The physiological arousal induced by the test batteries were assessed via measures of heart rate and heart rate variability (LF/HF ratio).

**Results:**

Satisfactory test-retest reliability and convergent validity with Cogstate attention and decision-making subtests and divergent validity in visual processing speed measures were observed. CONVIRT also increased heart rate and LF/HF ratio, which may better approximate participant arousal levels in their workplace.

**Conclusions:**

CONVIRT may be a reliable and valid tool to assess elements of cognition and CNS disruption. The increased ecological validity may also mean better informed ‘return-to-play’ decisions and stronger industry acceptance due to the real-world meaningfulness of the assessment. However, before this can be achieved, the sensitivity of the CONVIRT battery needs to be demonstrated.

## Background

Concussion amongst sportspersons is a worldwide concern due to both its prevalence and relationship with lifelong disease states [1]. Consequently, there has been substantial development in concussion management programs for athletes who play sports where the risk of concussion is high. Such strategies include recognition of risk factors, removal from play of injured athletes, careful management of return to play, and rule changes and education. In these strategies, the accurate assessment of cognition is crucial to both identifying the central nervous system (CNS) dysfunction that follows concussion and its resolution with time as athletes are managed toward their return to play [2]. Of all sportspeople, jockeys have the highest rate of concussion and the highest fatality rate (per minute of participation [3];). For example, jockeys fall 1 out of every 240 rides [4] and are 5 times more likely than an Australian Rules footballer to experience a concussion [5]. Therefore, concussion management programs that use cognitive assessment have been established in this group [6].

While multiple cognitive tests have been validated for use in concussion management programs, as newer research and emerging technologies provide important information and capabilities to guide refinement, there remains a need to continue to improve such assessment. For example, recent studies have found that in addition to cognition, careful analyses of ocular motor functions may provide insight into concussion related CNS dysfunction [7, 8]. In the technology domain, consumer grade virtual reality Head Mounted Displays (HMDs) have recently emerged providing the ability to readily immerse users in high quality virtual environments [9]. Eye-tracking, while not new to research studies, is now starting to be integrated into virtual reality headsets providing new opportunities in a range of domains.

There are three different types of ocular movements. Smooth pursuits are slow, voluntary, controlled movements that track a moving stimulus via speed and fixation [10]. Vergence movements are simultaneous and maintain fusion on objects near or far [11]. Saccades are rapid, ballistic eye movements that shift the gaze to new areas of interest and help bring a target into focus [12]. When a stimulus suddenly appears in the visual field, saccadic movement occurs voluntarily or reflexively [13]. Saccades require complex coordination of neural circuity in different brain regions (i.e., frontal lobe, basal ganglia, cerebellum), and therefore, act as a sensitive indicator of potential dysfunction in these areas [12]. Measurement of saccadic movement may be a sensitive tool for assessment of functional and structural cognitive impairment post-concussion which is lacking in clinical assessment of cognitive abilities [14]. Finally, compared to standard tests of cognition, such measurements are less likely to be influenced by individuals’ intellectual abilities, self-report, fatigue and/or practice effects [11, 15, 16].

Saccadic eye movements are associated with visual attention, visual discrimination [17, 18], working visual memory, decision-making [19], and visual processing speed [20]. Given these areas of cognition are often assessed in concussion management protocols, a measure of saccadic movement could enhance the sensitivity of such concussion assessments [11, 13, 21].

Recent research has highlighted that eye-tracking technology may be a useful adjunct to standard neuropsychological tests for mild traumatic brain injury (mTBI), but endeavours to include the technology have been limited by an inability to get such technology to function reliably and validly beyond laboratory settings [14]. Laboratory based equipment is cumbersome, involves adjusting camera positions and chin rests and as such, may compromise the reliability of such assessment. Recent advances in camera-based eye-tracking technology within wearable headsets however, have led to high precision tests that are reliable and valid [22, 23]. Using eye-tracking technolgy within a virtual-reality environment adds a further step towards ‘normalising’ the experience of such testing for the user.

While the scientific principles that guide concussion management programs are consistent across the different sports in which they are used, understanding better the specific context of cognitive testing within individual sports may result in a greater sensitivity to concussion related cognitive impairment. Current virtual-reality technologies enable a high level of visual and auditory immersion in a particular environment and task. As such, concussion testing that incorporates virtual-reality as well as the specific sporting content, may improve user acceptance and foster better adherence and compliance as athletes can better appreciate the relevance of the assessment to the optimal execution of their own sport. In the context of neuropsychological testing this principle is termed ecological validity. Specifically, this form of ecological validity is known as verisimilitude – which refers to the similarity of the instrument to relevant environmental behaviours [24].

We have developed a virtual-reality (VR) concussion assessment battery ‘CONVIRT’ that assesses components of attention, decision-making, and visual processing speed using assessments of manual simple and choice reaction time and saccadic reaction time. CONVIRT has the user complete the cognitive tests while riding a horse during a horserace in a virtual environment. VR paradigms have been shown to elicit heightened physiological arousal [25, 26]. When this information is considered alongside research which suggests that higher physiological arousal during cognitive testing was associated with poorer performance among jockeys [27] and students [28], it may be important to assess cognition when the athlete’s physiological arousal better approximates that required during their sport. Additionally, CONVIRT incorporates standard ‘distractors’ such as spectator sounds and movement of horses within the dynamic testing environment. These distractions are however, held constant across testing sessions.

The development of new measures for use in clinical settings must begin with understanding their performance in laboratory conditions. These studies focus on establishing psychometric test characteristics such as reliability and validity [29]. In addition, for cognitive tests designed to be given repeatedly it is crucial to know their stability over time. Furthermore, because the use of tests to manage CNS injury in the area of interest requires strong understanding of how performance may change (or ideally remain stable) in the absence of any true CNS impairment or CNS injury, it is necessary in the first instance that such estimates be obtained from healthy adults tested at intervals where the potential for any true or important changes in CNS state (for example, arising from fatigue, drug use, pain) are very small. Consequently, the aim of this study was to test the reliability of CONVIRT assessments over short retest intervals and also to assess convergent (simple and choice-reaction time) and divergent validity (saccadic reaction time) with comparable neuropsychological tests of concussion. In this study we used a sample of university students to assess the psychometric properties of CONVIRT. Once these properties are demonstrated we plan to test the sensitivity of the CONVIRT battery using a pre-post prospective study of concussion with a sample of jockeys. Finally, we aimed to determine if compared to the standard computer-based testing, CONVIRT elicited higher physiological arousal.

## Methods

### Participants

Participants (*N =* 165, females = 84) were Australian university students who were approached using a script and invited to engage in the research project. Participants (*Mage* = 22.91, range 18–34 years, *SD* = 3.50) were included if they were currently full-time students (academic stress assessed in separate study) and could read English, and excluded if they were currently unwell, had ongoing health problems, considered themselves physically fragile, or had received a concussion in previous 6 months. Given the student sample was healthy, with a comparable age-range, free from concussion, and similar to jockeys [27], are known to be highly stressed [28, 30], they served as an ideal proxy sample. Based on a power analysis for biserial correlation using G*Power 3 with a conservative medium effect of ρ = .30 (Falleti et al. [31] report very high effect sizes with a similar design), and power set at .80, an N of 64 was required to detect an association at *p =* .05*.* The sample size was larger than required for the present study, as some of the data will be used to answer other research questions where more statistical power is required. Participants (mean BMI = 24.98, *SD* = 5.32) provided written informed consent in line with institutional ethics (HEC S17–117) and were compensated for their time with a double cinema voucher.

## Materials

### The Cogstate battery

Three computerised tests from the Cogstate battery (Cogstate Research software; Version 6; Cogstate Limited, 2011) were included; the Detection task (DET), the Identification task (IDN), and the Groton Maze Chase test (GMCT) and administered using a 14-in. laptop. The individual tasks are designed to test a specific area of cognition over repeated sessions [32] and are routinely used to assess change post-concussion in elite, contact sports to help inform return to play decisions [33, 34].

#### Attention

The DET assesses psychomotor function through a simple reaction time paradigm. The participant is presented with a playing card that is face-down in the centre of the computer screen. The playing card is then turned face up and the participant must press the appropriate response key as quickly as possible after the card flips. The DET task ends after 35 correct responses. In healthy participants, the average completion time for this task is 3 min.

#### Decision making

The IDN is a choice reaction time test that measures psychomotor speed and decision making. The participant is again presented with a face-down card but is then instructed to indicate if the card is red or black as quickly as possible when the card is turned face up. The IDN task ends after 30 correct responses. In healthy participants the task takes approximately 3 min to complete.

#### Visual processing speed

The GMCT is a timed 30-s task designed to measure simple visuomotor processing speed. In this task, participants are required to follow a moving, coloured tile through a 10 × 10 grid, as quickly as possible using the computer mouse cursor.

The DET and IDN reaction times are recorded in milliseconds. These types of tests are known to produce positively skewed distributions and are routinely, statistically transformed using a natural logarithm to better represent a normal curve distribution [35, 36]. The GMCT score represents the average number of moves per second on the task. Therefore, low scores represent better performance on the IDN and DET tests and high scores represent better performance on the GMCT.

The Cogstate battery has demonstrated high test-retest reliability and limited practice effects at 10-min, one-week, and one-month intervals. For the DET task, intraclass correlations were 0.84 and 0.83 for the two 10-min intervals and 0.94 and 0.73 for the one-week and month intervals. The IDN task showed similar intraclass correlations, specifically 0.38, and 0.55, for the two 10-min intervals and 0.81 and 0.71 for the one-week and month intervals [36]. The DET and IDN have also demonstrated good convergent validity with conventional pencil and paper neuropsychological tests.

### The CONVIRT battery

**Fig. 1:**
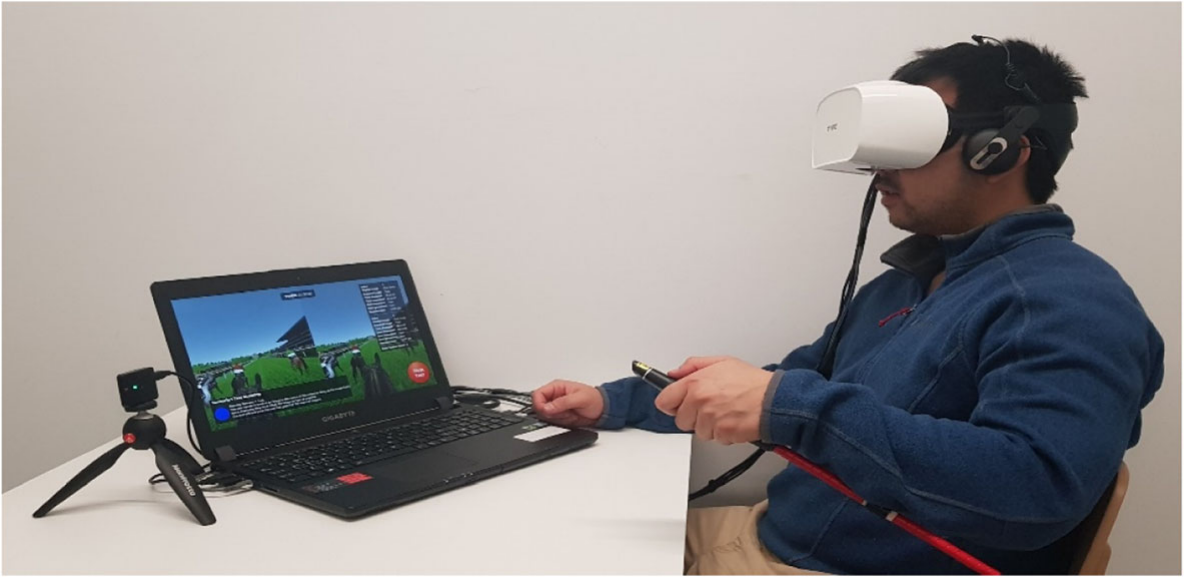
The CONVIRT battery set-up. The Head Mounted Display (HMD) has a 2560 × 1440 WQHD OLED screen with a refresh rate of 70 Hz (i.e., frames per second) that provides the visual display 1280 × 1449 to each eye. The HMD provides a field of view of 100 degrees vertically and 88 degrees horizontally. The eye tracking unit embedded in the FOVE HMD has a tracking accuracy of less than 1 degree and a refresh rate of 120 Hz (https://www.getfove.com). A custom developed riding crop provides a button for a subject to press while retaining the natural feel of a professional riding crop. The riding crop is modified in such a way that the subject would hold it similar to reality and their thumb would rest on a push button. The riding crop connects wirelessly to the laptop and subjects press this button to interact with the CONVIRT application

The CONVIRT battery comprises the CONVIRT VR application, FOVE 0 Eye Tracking VR Headset, and customised riding crop with button and wireless connectivity all running through a gigabyte P35 laptop (Fig. 1).

The FOVE 0 Eye Tracking VR Head Mounted Display (HMD) provides built in eye tracking functionality. The HMD displays the virtual environment to the subject as well as tracking their head rotation and direction of their gaze. The position and orientation of the HMD is tracked by an Inertial Measurement Unit (IMU) internal to the HMD as well as by an external tracking camera.

The CONVIRT application runs on a Gigabyte P35 laptop. The laptop is running 64-bit Windows 10 Home OS, has an i7-6700HQ CPU, 16Gb of DDR4 RAM and a GEFORCE GTX 1070 graphics card. The laptop is capable of running the CONVIRT application and the FOVE headset at 70 frames per second (FPS) for the display and 120 fps for eye tracking sensors, in order to provide the most accurate results. These are the highest update rates for the FOVE headset.

The CONVIRT VR application is built in the Unity gaming engine and runs on the 64-bit Windows 10 operating system. The application places the user on a virtual horse running on a racecourse modelled to be similar to a professional horse racing track. The tool consists of three tests. Each test presents floating shapes in front of the user to which they must respond to in a manner dependant on the test being conducted. The shapes are positioned at different points along an invisible 180-degree arc in front of the virtual horse and give the experience of being in extra-personal space. The targets (and distractors) are on a vertical plane in front of the user at a distance of 1.5 m from the user’s head, and the arc centrepoint is directly in front of the user’s head, and the arc has a radius of 0.5 m.

**Fig. 2:**
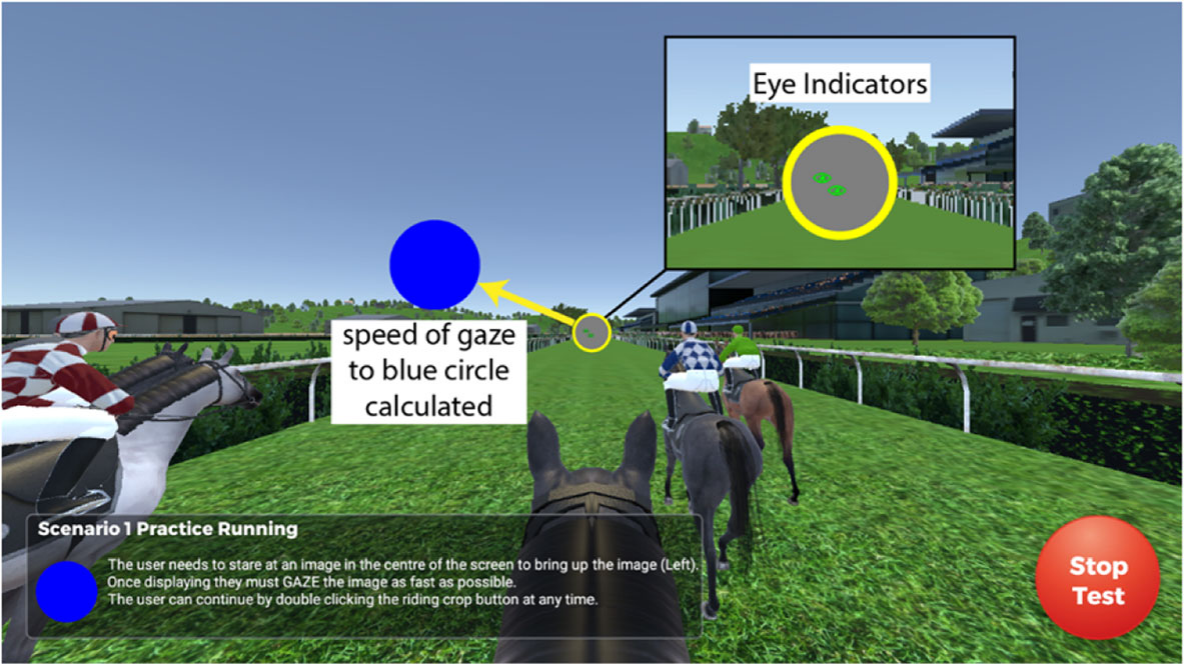
The environment/experience of the test of saccadic reaction time. The shapes have width and height of 0.4 m and are presented to the user at a distance of 1.5 m away within the virtual world. This results in a visual angle, i.e. angular size, of approximately 13 degrees for each of the target stimuli (range = 143 degrees horizontally, and 71.5 degrees vertically which is smaller than the geometric field of view in the FOVE HMD)

The design of the environment in all tests is suitable for those who are colour-blind (Fig. 2). All tests have instructions embedded to the user’s view and have a practice trial before each test. Participants are seated in a fixed chair during testing.

#### Pilot testing

The CONVIRT battery was trialled with professional jockeys (*n* = 7) and jockey coaches (*n* = 3) to ensure the virtual environment mirrored that of a professional horserace. Slight modifications to the gait of the virtual horses were recommended and then implemented. None of the jockeys or coaches reported any nausea or motion sickness. Careful consideration was given to following best practice design with a specific focus on reducing sensory-conflict and latency that are well known contributors to sickness when using VR [37]. To minimise impact from sensory conflict between vision and vestibular systems, the horses and user move along the track at a constant velocity of 20 km/h and accelerations are avoided. Modern headsets have significantly decreased latency times and in the design of CONVIRT, consideration was given to allow the frame rate of the virtual experience to run at the maximum possible framerate allowing the headset to have minimal delays (lowest latency) in updating the visual information displayed to the user. The events inside the VR experience (e.g. bobbing due to horse running motion) do not alter the height of the user’s view within VR while riding down the virtual track. The user however can turn their head to view the track around them, or also lower or raise their head while sitting in the chair. This motion is limited to the ability of the user to hunch down or stretch up and is rather minimal relative to the sizes of the objects in the virtual scene, as well as the distance from the user’s head to the invisible plane where the targets (and distractors) appear. Early feedback from jockeys and their coaches confirmed that CONVIRT adequately represented a horse race experience.

#### Visual processing speed

The first test is an eye tracking test that measures a subject’s saccadic reaction time (SAC). When the test starts the subject needs to stare at a grey circle, positioned in the centre of the above mentioned 180-degree arc, for a duration of 2 sec. After this time the grey circle will disappear, and a blue circle will appear somewhere on the arc (Fig. 2). The subject needs to look at the blue circle with both eyes, as quickly as possible. When the subject’s gaze converges on the blue circle it will ‘explode’ (visual representation of disintegration accompanied by auditory explosion sound) and then disappear and the grey circle will reappear and the process is repeated when the participant holds their gaze on the grey circle for 2 sec. This process continues until the subject has looked at 35 blue circles. The position of blue circles presented at 13 degrees in the left hemisphere of the arc, are mirrored in the right hemisphere (17 each side). One blue circle is presented in the midline above the grey circle. The time it takes for the subject to move their gaze from the grey circle to the blue circle is recorded and additionally, we measured the time taken to move 50% towards the target. This portion of the saccade will capture the response latency (the time taken to initiate the saccade), which is approximately 200 ms. [38] and a component of acceleration towards the target. We chose not to use the complete time to converge on the target as this would incorporate a variety of more complex neural processes including deceleration prior to reaching the target, adjustments to improve gaze accuracy, and the fact that saccades only account for approximately 90% of the movement between the eye and the target [38]. It was this measure of SAC that was used in all subsequent analyses.

#### Attention

The second test (DET^VR^) evaluates a subject’s manual simple reaction time to a stimulus. An orange triangle randomly appears in front of the user at a point on the arc. Once the user detects a triangle, they need to press the button on the riding crop resulting in the triangle disappearing for differing durations (ranging from 1 s. to 2.37 s.). Similar to the Cogstate DET test, the test lasts for 120 s and 35 triangles are displayed to the subject. The reaction time is recorded along with any presses of the push button when the triangle is not present, i.e., false positives.

#### Decision-making

The third test (IDN^VR^) assesses choice reaction time. This test is similar to the previous test in that the user must respond using the riding crop button to a shape appearing. The subject needs to respond to an orange circle appearing and must not respond to a blue triangle or blue circle appearing. Similar to the Cogstate IDN test, the test lasts for 120 s and in that time 31 shapes are displayed to the subject. The reaction time of the subject is recorded along with any incorrect responses.

### Heart rate variability (HRV)

The measures of heart rate (HR) and HRV were used to assess if the CONVIRT experience was more physiologically arousing than the seated computer-based Cogstate testing. Heightened sympathetic arousal on both HR and HRV measures is indicated by higher scores. HRV is an indirect measure of the autonomic nervous system and may reflect the push-pull relationship between the sympathetic and parasympathetic nervous system [39]. The present study measured the low frequency (LF) to high frequency (HF) ratio of spectral HRV. An increased LF/HF ratio is associated with increased stress [40]. It has been suggested that a higher LF/HF ratio reflects sympathetic dominance while decreases correspond to parasympathetic dominance [41]. The measures of heart rate (beats per min) and LF/HF were used to assess if autonomic arousal differed during CONVIRT and Cogstate testing. Consecutive RR intervals were recorded using a wireless heart rate monitor (RS800CX; Polar, Finland). The Polar heart rate monitors have been shown to produce electrocardiography comparable measures of RR-interval with derived time domain HRV [42]. After removing non-sinus RR-intervals, consecutive RR-intervals were analysed using customised commercial software (LabVIEW 2016; National Instruments, UK) to determine short-term HRV in the time domain for 5-min. Epochs in user-selected time blocks.

### Procedure

Upon arrival to the lab, participants provided informed consent. They were then given instructions on how to fit the HR monitor and privacy to do so. Once fitted, participants completed a questionnaire pack (data not reported in this study), which included demographic information, as well as height and weight to enable to calculation of body mass index (BMI). BMI is routinely entered as a covariate in analyses involving HRV. Recording of HR and the LF/HF ratio began immediately and continued throughout the experiment.

#### Baseline phase (*M = 8.58 mins, SD = 2.61 mins, range = 5–24 min*)

Participants sat at a table with the equipment for the CONVIRT and Cogstate tests placed in front of them. Participants completed their questionnaires. The baseline period was used as a means of collecting data, but also to minimise the impact of anticipatory arousal on test performance. Strong anticipatory physiological responses before Cogstate testing has been observed in similar studies using these cognitive measures with measures of HRV [28].

#### CONVIRT 1 (*M = 15.45 mins, SD = 4.25 mins, range = 11–25 min*)

Participants were fitted with the FOVE 0 Eye Tracking VR Headset and given the customised riding crop. Participants completed the CONVIRT tests in the order they are presented above. Each test involved the participant receiving instructions on screen and the opportunity to complete a practice trial to ensure they were familiar with the assessment requirements and the use of equipment before the participant completed the test.

#### Cogstate (*M = 8.08 mins, SD = 1.72 mins, range = 5–17 min*)

The participants then completed the Cogstate battery in a consistent order (i.e., DET then IDN then GMCT). Participants were provided with on-screen instructions and practice trials before each task to ensure they understood the requirements of each task. Practice trials are routinely conducted before each task in the Cogstate battery [36].

#### CONVIRT 2 (*M = 12.40 mins, SD = 3.22 mins, range = 6–27 min*)

Participants were directed to complete the CONVIRT tests for a second time. The procedure was the same as the CONVIRT 1 phase.

### Data analysis

Repeated measures MANCOVA (age, BMI covariates) was used to assess if participants differed in heart rate or HRV when using the CONVIRT compared to Cogstate tests. Intra class correlation (ICC) was used to assess the test-retest reliability of CONVIRT and convergent and divergent validity with comparable Cogstate measures. Convergent and divergent construct validity were examined using Pearson’s correlation coefficient and were evaluated using the guidelines (correlation levels: negligible = 0.00–0.19, weak = 0.20–0.39, moderate = 0.40–0.59, strong = 0.60–0.79, very strong = 0.80–1.00). Convergent and divergent validity was classified if *r* > 0.70 or <  0.30 respectively [43]. ICC values were interpreted as > 0.75 = excellent, 0.40 to 0.75 = fair to good, and <  0.40 = poor [44].

## Results

### Data management

In line with the transformed Cogstate variables, a natural logarithm transformation was applied to the three CONVIRT measures. There was no missing data and the assumptions for parametric testing for ANCOVA and intra class correlation were satisfied [45].

### Analyses

The mean and standard deviation for each of the cognitive tests (Table 1) and measures of HR and LF/HF across each phase of the study (Table 2) are reported. A repeated measures MANCOVA was used to assess if participant HR or the LF/HF measures of autonomic reactivity differed between CONVIRT and Cogstate testing paradigms after controlling for age, gender and BMI.

**Table 1:** Average speed of reaction to each Cogstate and CONVIRT test

	Variable	*M*	*SD*
Cogstate	DET	2.53	.08
	IDN	2.68	.06
	GMCT	1.67	.29
CONVIRT	DET^VR1^	2.44	.06
	DET^VR2^	2.45	.05
	IDN^VR1^	2.56	.06
	IDN^VR2^	2.55	.06
	SAC 1	2.44	.09
	SAC 2	2.43	.06

Note. For the GMCT the mean values represent moves per sec. The mean values for all other tests were in milliseconds and have been transformed by natural logarithm

**Table 2:** HR and LF/HF ratio Means and Standard Deviations during each Experimental phase

Measure	Experimental sequence			
	Baseline (1) *Mean (SD)*	CONVIRT (2) *Mean (SD)*	Cogstate (3) *Mean (SD)*	CONVIRT (4) *Mean (SD)*
HR (bpm)	87.28 (18.98)	81.38 (12.42)	78.98 (11.75)	80.51 (12.84)
LF/HF (ms)	4.2 (3.1)	3.5 (2.8)	3.3 (2.2)	4.5 (5.7)

The MANCOVA revealed differences in HR and LF/HF across the CONVIRT and Cogstate testing *F*(4, 160) = 10.59, *p* < .001. Using a Bonferroni adjustment, the criterion alpha level was .025 for the two comparisons made for each measure of physiological arousal. Participants had higher HR and LF/HF ratios in all cases except for the LF/HF comparison between Cogstate and the first CONVIRT test, although this comparison trended in the anticipated direction (Table 3). The effect sizes were all small by Cohen’s convention where d values of .20, .50 and .80 correspond with small moderate and large effects, respectively [46].

**Table 3:** Comparison of HR and LF/HF ratio between Cogstate and CONVIRT 1 and CONVIRT 2

	Comparison	*Cohen d*	*p* value
HR	Cogstate-CONVIRT 1	.24	<.001
	Cogstate-CONVIRT 2	.15	.007
LF/HF	Cogstate-CONVIRT 1	.08	.241
	Cogstate- CONVIRT 2	.28	.005

The final set of analyses assessed the test-retest reliability (average 8.08 min. apart) of the CONVIRT measure and the convergent validity of the CONVIRT tests with comparable Cogstate measures (Table 4).

**Table 4:** Intra Class Correlations between each test for CONVIRT 1 and 2, and Cogstate

*Comparison*	*r*	*p*
DET^VR 1^ - DET^VR 2^	.90	< .001
DET^VR 1^ - DET (Cogstate)	.59	< .001
DET^VR 2^ - DET (Cogstate)	.58	< .001
IDN^VR 1^ - IDN^VR 2^	.88	< .001
IDN^VR 1^ - IDN (Cogstate)	.73	< .001
IDN^VR 2^ - IDN (Cogstate)	.75	< .001
SAC 1 - SAC 2	.76	< .001
SAC 1 - GMCT (Cogstate)	.26	.027
SAC 2 - GMCT (Cogstate)	.26	.022

The approximate 10 min. Test-retest reliability of the CONVIRT measures were excellent for the DET and IDN and SAC measures [44]. The convergent validity of the CONVIRT DET and IDN was acceptable with the Cogstate DET and IDN tests (both > 0.70), and the low association between the SAC and GMCT (< 0.30) tests [43] suggests divergent validity between these measures (Table 4).

## Discussion

The CONVIRT tests demonstrated high test-retest reliability and satisfactory convergent and divergent validity with related subtests from the Cogstate battery. CONVIRT also elicited greater physiological responses when compared to the Cogstate battery.

Establishing the test-retest reliability of cognitive tests used to assess sport-related concussion is important given that these tests are used to compare baseline performance against post-concussion performance. The DET and IDN task have demonstrated satisfactory levels of reliability with intraclass correlation coefficients reaching .60 or above [36, 47], which is said to be the accepted minimum for making clinical decisions [48]. The CONVIRT tool demonstrated even larger intraclass correlation coefficients with each task exceeding .75, which is typically considered the acceptable level for test-retest reliability [48, 49].

CONVIRT also demonstrated satisfactory convergent validity with two of the subtests correlating with the conceptually matched Cogstate subtests. Specifically, the DET^VR^ and the IDN^VR^ at both timepoints were moderately correlated with the DET and IDN, respectively. This is in line with what would be expected given that both tasks are assessing attention and decision-making via reaction times.

The SAC and GMCT tasks showed only a small correlation. This is likely due to the substantive differences in measurement of visual processing speed. The SAC uses eye tracking technology to assess saccade reaction time. In contrast, the GMCT infers visual processing speed from the participants ability to follow a target using a computer mouse cursor. Measures of saccadic response are showing promise in being able to effectively distinguish between those with mTBI and those without [50]. Measures of saccadic reaction time involve attention and cognition [50] and incorporate diffuse networks across both cortical and subcortical structures [51]. Assessments of saccadic reaction time may be more sensitive to subtle changes in a number of these pathways than more traditional cognitive assessments. Our SAC measure shows divergent validity from the GMCT and given technological advances with HMD’s is likely to be comparable with laboratory-based eye-tracking equipment. Nevertheless, research is required to confirm this assumption.

In the current study, participants using CONVIRT recorded increased HR and LF/HF ratios compared to Cogstate. CONVIRT may be more engaging for participants and better approximate the physiological responses that occur during a horserace. Indeed, VR paradigms have been shown to have higher ecological validity than standard neurocognitive tests [52] and are associated with increases in physiological arousal [25, 26]. Having higher ecological validity may promote stronger industry acceptance of the tool given its real-world application and may also lead to better informed return-to-play decisions. Some may question whether the differences in physiology across phases were driven by habituation effects. If this were the case, we would anticipate a linear trend post-baseline. However, on both HR and LF/HR measures a decrease in these measures is recorded during Cogstate followed by an increase when participants return to the second CONVIRT assessment.

The use of virtual-reality environments has previously been associated with feelings of nausea in some participants [53]. In the present study, we did not formally assess if participants experienced nausea. However, CONVIRT was designed specifically to minimise nausea occurring using best practice and design guidelines for VR including a specific focus on minimising vision-vestibular system mismatch by using constant velocity and avoiding accelerations and keeping the headset framerate at maximum (and headset visual latency at the minimum). We can also report that none of the participants reported nausea or asked the testing to be stopped. These experiences are consistent with what was observed in our pilot testing with jockeys.

The findings also have implications for university students. Students are highly stressed [28, 30] with research showing that higher physiological arousal and perceived stress interact to decrease performance on the Cogstate tests for both students [28] and jockeys [27]. The CONVIRT tool, which is more arousing than the Cogstate battery, may offer additional insights into these relationships. For university students, performance on academic tasks may be compromised by stress. In future research, stress-reduction interventions may be paired with CONVIRT performance to assess their efficacy in improving attention and decision-making.

The current study demonstrates the test-retest reliability and convergent validity of the CONVIRT tool. However, future studies are planned to assess the test-retest reliability over longer time-intervals and to test the sensitivity of this battery in detecting changes in cognitive abilities. Additionally, in the present study, visual processing speed was measured using the time elapsed to 50% of the total distance to the target stimulus. We have outlined that capturing data from the final 50% of the ocular movement towards the target may include more complex neuronal processes (e.g., deceleration, accuracy adjustments etc.). Our focus was on the speed of saccade and not accuracy, and this aligns with decisions made for other neurocognitive assessment batteries [32]. That said, in future research, an analysis of the final segments of the gaze-to-target time and measures of vergence may also be useful in assessing CNS performance post-concussion. Finally, we did not assess the time lag associated with using the Bluetooth device for measures of simple and choice reaction time in the CONVIRT battery, we will run this experiment in the future. However, as the speed and variability of performance on these tests was the same or lower than the Cogstate computer-based measures (Table 3), this is unlikely to be a substantive issue.

The CONVIRT test offers several advantages over computer-based testing. For example, the dynamic environment resembles the workplace, but it requires less interaction with the tester, ensures stable light and sounds, and as the SAC test assesses a mixture of exogenous and endogenous processes, the impact of practice effects is reduced.

## Conclusions

Jockeys are at very high risk of concussion and decisions about when they are safe to return to ride post-concussion are guided by measures of their cognitive function. However, as the current measures of cognitive performance for jockeys have low ecological validity, this may compromise the decisions made using such measures.

Ensuring that jockeys have regained their pre-concussion cognitive abilities prior to re-engaging with the sport is vital in protecting them from a heightened risk of falling [4] and injury. The CONVIRT battery appears to incorporate reliable and ecologically valid measures of attention, decision-making and visuomotor processing speed, but future assessments are required to assess the sensitivity of the tool- and also if these findings generalise to a sample of jockeys. Our findings suggest that the added visual and auditory distractors in the virtual environment improve the ecological validity of the testing without impacting the reliability or construct validity of measurements. Given that these high levels of reliability and validity can be obtained in virtual reality without inducing sickness suggests that CONVIRT may prove to be an important adjunct to standard computerised neuropsychological testing.

## Data Availability

The data for all analyses are available in the Open Science Framework repository at https://osf.io/g8p5t/

## Abbreviations

LF: Low frequency
HF: High frequency
HMD: Head mounted display
CNS: Central nervous system
mTBI: Mild traumatic brain injury
VR: Virtual reality
DET: Detection test
IDN: Identification test
GMCT: Groton maze chase test
SAC: Saccadic reaction time
DET^VR^: CONVIRT detection test
IDN^VR^: CONVIRT identification test
IMU: Inertial measurement unit
HRV: Heart rate variability
FPS: Frames per second
BMI: Body mass index
ICC: Intraclass correlation coefficient
BPM: Beats per minute
ms: Milliseconds
HR: Heart rate
